# Calcineurin inhibitors in systemic sclerosis – a systematic literature review

**DOI:** 10.1177/1759720X221092374

**Published:** 2022-05-19

**Authors:** Nina N. Hofmann, Robert A. Ambühl, Suzana Jordan, Oliver Distler

**Affiliations:** Department of Rheumatology, University Hospital Zurich, University of Zurich, Zurich, Switzerland; Department of Rheumatology, University Hospital Zurich, University of Zurich, Zurich, Switzerland; Department of Rheumatology, University Hospital Zurich, University of Zurich, Zurich, Switzerland; Department of Rheumatology, University Hospital Zurich, University of Zurich, Schmelzbergstr. 24, Zurich 8091, Switzerland

**Keywords:** calcineurin inhibitors, cyclosporin A, systemic sclerosis

## Abstract

**Objective:**

To review treatment effectiveness and adverse events of calcineurin inhibitors (CNIs) such as cyclosporin A (CsA) and tacrolimus in patients with systemic sclerosis (SSc).

**Methods:**

A systematic literature search was performed on PubMed and Web of Science using the predefined keywords ‘systemic sclerosis’, scleroderma, cyclosporin*, and tacrolimus. Articles were eligible for inclusion, if SSc patients had been treated with CNIs and data on treatment effects were available.

**Results:**

This systematic literature review identified 37 papers (19 case reports, 15 case series, 2 controlled studies, and 1 retrospective study) including 134 SSc patients treated with CNIs. In 34 of 37 papers, CsA was used. An improvement of skin fibrosis was observed in 77 of 96 (80.2%) patients using a wide variety of outcome measures and dose regimes. Both controlled studies showed significant improvements, one using a historical control group and one using a no-treatment control group. Improvement in pulmonary function tests (PFTs) occurred in 67.9% (19/28) of the patients who had reduced PFTs at baseline. In 58 (43.3%) cases, adverse renal events were reported, of which 7 (5.2%) were severe such as scleroderma renal crisis (SRC), CsA-associated nephropathy, or death by renal insufficiency. Adverse events led to dose reduction, treatment interruption, or withdrawal in 39 of 134 (29.1%).

**Conclusion:**

In this systematic literature review, signals for potential effectiveness of CsA for skin and pulmonary fibrosis were found, but the evidence level of the identified studies was too low to allow robust conclusions. Randomized controlled double-blind trials are needed to conclude on the effectiveness of CNIs in SSc. Renal toxicity of CNIs was confirmed in this review and needs to be considered in the design of such studies.

## Introduction

Systemic sclerosis (SSc) is a rare, potentially lethal autoimmune connective tissue disease, characterized by fibrosis affecting the skin and a variety of internal organs.^1^ Very early disease manifestations are Raynaud’s phenomenon (RP), SSc-specific autoantibodies, and characteristic microvascular changes detected by nailfold capillaroscopy.^2^

During the course of the disease, the majority of SSc patients develop skin fibrosis, measured by the modified Rodnan skin score (mRSS). Skin thickening is one of the major aspects of the classification criteria for SSc.^3,4^ Improvement in skin thickness correlates with better functional outcome and lower morbidity and mortality.^5,6^

Interstitial lung disease (ILD) is detectable in >50% of dcSSc patients and in >30% of lcSSc patients, respectively.^7^ ILD and pulmonary hypertension (PAH) are the leading causes of SSc-associated deaths.^3,8^

In the past, the major organ complication was scleroderma renal crisis (SRC), associated with accelerated arterial hypertension and rapidly progressive deterioration of kidney function.^9^ Nowadays, most likely due to of the treatment with angiotensin converting enzyme (ACE) inhibitors, it is no longer the leading cause of death.^7,10^ Nevertheless, up to two thirds of cases require long-term dialysis and mortality remains high.^3^ To improve poor prognosis, early detection and immediate therapy are important.^11^

Treatment of SSc is challenging, due to the complexity and wide spectrum of disease manifestations. Today, treatment mostly consists of organ-specific management.^12^ The updated EULAR recommendations of 2017 and more recent organ-specific management guidelines provide an evidence-based overview concerning pharmacological treatment of SSc-related organ involvement.^13,14^ Symptomatic treatment led to improved clinical outcome over the years. However, established treatments for the overall disease process and fibrotic manifestations are still not available.^15,16^ Autologous hematopoietic stem cell transplantation (HSCT) showed promising results in several randomized controlled clinical trials, but its use might be limited due to increased mortality in the first year of treatment.^17^ Recently, based on the findings of the SENSCIS trial, nintedanib was approved for treatment of SSc-ILD in various countries.^18^ Nintedanib is a tyrosine kinase inhibitor leading to anti-fibrotic but also to anti-inflammatory effects.^19^ Moreover, the FDA approved the interleukin-6 receptor antibody tocilizumab for the treatment of SSc-ILD based on the focuSSced trial.^20^

Cyclosporin A (CsA) and tacrolimus (Tac), a group of immunosuppressive agents referred to as calcineurin inhibitors (CNIs), could be another treatment option for SSc patients.^21^ By forming a complex with either cyclophilin or FK506 binding protein (FKBP), they competitively bind to calcineurin, inhibiting translocation of the nuclear (transcription) factor of activated T-cells (NF-AT).^22^ This leads to a reduced transcription of cytokines and inhibition of T cell activation.^23^ Accordingly, CsA has been used to treat patients with SSc and reports with promising effects on skin fibrosis have been published. However, its use in clinical practice and its formal testing in large prospective randomized controlled clinical trials have been restricted by its potential renal toxicity and reports about the occurrence of SRC in patients treated with CsA. Therefore, the aim of this study was to provide a structured and unbiased review of potential effectiveness and toxicity of CNIs in SSc using a systematic literature analysis.

## Materials and methods

### Systematic literature search

A systematic literature search on PubMed and Web of Science was performed using the following two terms: (1) «systemic sclerosis» OR scleroderma and (2) cyclosporine* OR tacrolimus. Using the advanced search method, one query was added to the other by the word «AND». Filters were applied for language (English, German) and article type (case reports, clinical study, clinical trial, controlled clinical trial, historical article, randomized controlled trial, journal articles). All articles were included up to 31.12.2019. PRISMA guidelines were followed (the PRISMA Checklist is available as supplemental material). The searches were performed by two authors each and uncertainties were discussed.

### Inclusion criteria

In addition to the applied filters, preclinical experimental studies, abstracts, letters or congress reports were excluded. Reviews addressing SSc and CNIs were used to screen for additional primary research publications, but not for the primary analysis. Only papers with SSc patients treated with CNIs and available information on the outcome of CNI therapy on SSc disease manifestation were analysed. Therefore, studies of SSc patients receiving CNI therapy for reasons other than SSc (e.g. organ transplantation) were excluded if the effect on SSc was not specified. If this was the case, or if the article did not show the effects of CNI therapy in SSc patients, the reason for exclusion was labelled as ‘No specific analysis for SSc or CNIs’. In addition, articles were excluded, if they reported on other forms of scleroderma (e.g. morphea/ localized scleroderma). Inclusion and exclusion criteria were analysed by the investigators (NH, RAA, SJ, OD) independently. In case of uncertainties and discrepancies, consensus was obtained after discussion.

### Data extraction and analysis

Patient characteristics and treatment data of the included articles were collected, including gender, age, subtype, disease duration, disease manifestations, previous treatments, co-treatments, dosage and duration of CNI therapy as well as adverse events. Studies with co-treatment with other immunosuppressive agents including prednisolone (PSL) equivalents >10 mg/d were also listed and analysed separately.

When assessing disease manifestations, many different methods and scores were used for measurement (Table 2).

Different duration of treatments were reported. The most frequent definition was the time from initiation of therapy to the end of follow-up or discontinuation of CNI. If treatment was discontinued and re-administered, treatment periods were added. Time between dose reduction and complete stop of treatment was not included into calculation of therapy duration. In some studies, there was no clear information on therapy duration provided.

Because most of the identified articles were dealing with skin and lung fibrosis, we focused our effectiveness analysis on these two organ manifestations. Accordingly, all available baseline and follow-up measures for skin and lung fibrosis were extracted, and effectiveness as well as side effects of CNI treatment was collected.

## Results of the systematic literature search

**Figure 1.**
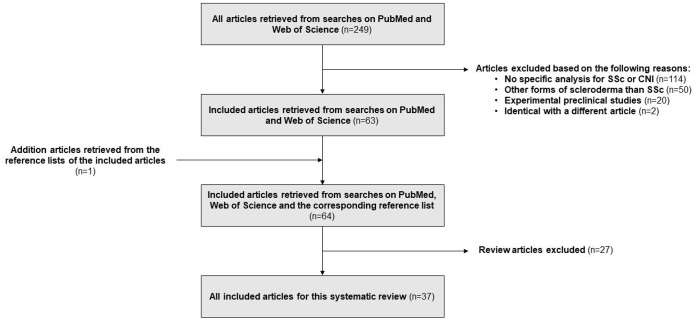
Literature review flow diagram.

From the systematic literature search, 249 articles were retrieved, from which 186 were excluded. Reasons for exclusion are listed in Figure 1. References of the remaining 63 papers were screened for additional articles, and one further article was found.^24^ Finally, 27 review articles were excluded, and the remaining 37 identified primary research articles were included into the analysis.^24–60^ The 37 articles consisted of 19 case reports, 15 case series, 1 open-label, randomized controlled trial with a no-treatment arm (group I iloprost *vs* group II iloprost with low-dose CsA), one open-label, prospective study with a historical control group (CSA group *vs* historical placebo group of the chlorambucil trial),^61^ and one retrospective study without control (patients received prednisolone (0.5 mg/kg/d) and tacrolimus; Table 1).

**Table 1.** List of the analysed publications with information on study type, control group, and applied drugs.

Publication	Study type	Control	CNI	Co-treatments^a^
al-Mayouf *et al.*^25^	Case report	No	CsA	
Amor and Dougados^26^	Case series	No	CsA	
Ando *et al.*^27^	Case report	No	CsA	PSL^LD^ (1/1)
Appelboom and Itzkowitch^28^	Case report	No	CsA	PSL^HD^ (1/1)
Basso *et al.*^29^	Case series	No	CsA	
Basso *et al.*^30^	Case report	No	CsA	
Casoli *et al.*^31^	Case report	No	CsA	
Chen *et al.*^32^	Case report	No	CsA	
Clements *et al.*^33^	Historically controlled trial	Yes	CsA	
Constantopoulos *et al.*^34^	Case report	No	CsA	PSL^HD^ (1/1)
Davies and Dunn^35^	Case report	No	CsA	
Denton *et al.*^36^	Case series	No	CsA	PSL^LD^ (1/3)
Filaci *et al.*^37^	Randomized controlled trial	Yes^b^	CsA	
Francès *et al.*^38^	Case series	No	CsA	
Gisslinger *et al.*^39^	Case series	No	CsA	PSL^LD^ (4/8)
Heickendorff *et al.*^40^	Case series	No	CsA	
Hider *et al.*^41^	Case report	No	CsA	AZA (1/1)
Ippoliti *et al.*^42^	Case series	No	CsA	
Ishida *et al.*^43^	Case report	No	CsA	
Knop and Bonsmann^24^	Case series	No	CsA	
Konma *et al.*^44^	Retrospective study	No	Tac	PSL^HD^ (11/11)
Mehregan and Su^45^	Case report	No	CsA	PSL^HD^ (1/1)
Merot *et al.*^46^	Case report	No	CsA	PSL^HD^ (1/1)
Morton and Powell^47^	Case series	No	CsA, Tac	PSL^LD^ (1/15)
Nunokawa *et al.*^48^	Case report	No	Tac	PSL^HD^ (1/1)
Patrick *et al.*^49^	Case series	Yes^c^	CsA	
Quartier *et al.*^50^	Case series	No	CsA	PSL^HD^, MTX (4/4)
Roch *et al.*^51^	Case report	No	CsA	
Tooze *et al.*^52^	Case report	No	CsA	
Vayssairat *et al.*^53^	Case series	No	CsA	
Wörle *et al.*^54^	Case series	No	CsA	
Yamasue *et al.*^55^	Case report	No	Tac	PSL^LD^ (1/1)
Yocum and Wilder^56^	Case report	No	CsA	
Zachariae and Zachariae^57^	Case series	No	CsA	
Zachariae *et al.*^58^	Case series	No	CsA	
Zachariae *et al.*^59^	Case report	No	CsA	PSL^HD^ (1/1)
Zentilin *et al.*^60^	Case report	No	CsA	

a Co-treatments were frequently used and are listed if other immunosuppressive agents including corticosteroids (PSL^HD^ = high-dose prednisone (>10 mg/d), PSL^LD^ = low-dose prednisone (⩽10 mg/d)) have been applied.

b The control group received iloprost, the treatment group iloprost, and CsA.

c Healthy control group, but only to compare sIL-2R levels.

### Baseline patient characteristics

The 37 studies involved 134 SSc patients treated with CNIs. Patient baseline characteristics are summarized in Table 2. In 15 studies, the classification of SSc was made in accordance to the ACR criteria. Regarding subtype of the disease, 71 patients had dcSSc (53%), 23 lcSSc (17.2%), of which four were diagnosed as having the so-called CREST syndrome (calcinosis, RP, esophageal dysmotility, sclerodactyly, and telangiectasia), and three had overlap SSc (2.2%). Thirty-seven patients (27.6%) had no subtype specified. Out of the 134 treated patients, 90 were female (67.2%) and 24 male (17.9%). No information on sex was provided in the remaining 20 patients (14.9%). The patients were 8–78 years old (mean ± SD 45.1 ± 15.6) and had an average disease duration ranging from 0 to 30 (mean ± SD 5.8 ± 6.2) years.

**Table 2.** Patients characteristics at baseline.

Study	Pat (F/M)	Disease subtype	Mean SSc duration (Min–Max)	Organ involvement
al-Mayouf *et al.*^25^	1 (1/0)	O	6 years	Skin (1), lung (0), RP (1), oesophagus (0), heart (0)
Amor and Dougados^26^	2 (2/0)	D	N/A	Skin (2), lung (2), RP (2), oesophagus (2), heart (-)
Ando *et al.*^27^	1 (1/0)	D	0 years	Skin (1), lung (1), RP (-), oesophagus (-), heart (-)
Appelboom and Itzkowitch^28^	1 (1/0)	N/A	~0.42 years	Skin (1), lung (1), RP (0), oesophagus (1), heart (1)
Basso *et al.*^29^	9 (7/2)	D §	9.6 years (3–20 years)	Skin (9), lung (7), RP (-^a^), oesophagus (8), heart (0)
Basso *et al.*^30^	1 (1/0)	N/A	4 years	Skin (1), lung (1), RP (1), oesophagus (1), heart (-)
Casoli *et al.*^31^	1 (1/0)	N/A	0.5 years	Skin (1), lung (1), RP (1), oesophagus (-), heart (-)
Chen *et al.*^32^	1 (0/1)	D	0.5 years	Skin (1), lung (-), RP (1), oesophagus (-), heart (-)
Clements *et al.*^33^	10 (8/2)	D (9), L (1) §	~1.9 years (≤5 years)	Skin (10), lung (7), RP (-), oesophagus (7), heart (4)
Constantopoulos *et al.*^34^	1 (1/0)	D	9 years	Skin (1), lung (1), RP (0), oesophagus (-), heart (-)
Davies and Dunn^35^	1 (0/1)	D	2 years	Skin (1), lung (0), RP (1), oesophagus (1), heart (0)
Denton *et al.*^36^	3 (3/0)	D	N/A (2–3 years)	Skin (3), lung (1), RP (2), oesophagus (1), heart (-)
Filaci *et al.*^37^	10 (N/A)	D (8), L (2) §	N/A (<2 years)	Skin (10), lung (-^b^), RP (10), oesophagus (-^b^), heart (-^b^)
Francès *et al.*^38^	4 (N/A)	D §	N/A (<2.5 years)	Skin (4), lung (2), RP (-), oesophagus (4), heart (-)
Gisslinger *et al.*^39^	8 (4/4)	N/A §	4.5 years (1–8 years)	Skin (8), lung (7), RP (-), oesophagus (8), heart (8)
Heickendorff *et al.*^40^	6 (N/A)	N/A §	N/A	Skin (6), lung (-), RP (-), oesophagus (-), heart (-)
Hider *et al.*^41^	1 (1/0)	L	3 years	Skin (1), lung (1), RP (1), oesophagus (-), heart (0)
Ippoliti *et al.*^42^	5 (4/1)	N/A §	N/A	Skin (5), lung (4), RP (-), oesophagus (4), heart (1)
Ishida *et al.*^43^	1 (1/0)	D §	N/A	Skin (1), lung (1), RP (1), oesophagus (1), heart (-)
Knop and Bonsmann^24^	3 (2/1)	N/A §	N/A (>3 years)	Skin (3), lung (2), RP (3), oesophagus (-), heart (-)
Konma *et al.*^44^	11 (11/0)	D (7), L (4)	N/A	Skin (11), lung (11), RP (-), oesophagus (-), heart (-)
Mehregan and Su^45^	1 (1/0)	N/A	2.5 years	Skin (1), lung (-), RP (1), oesophagus (-), heart (-)
Mérot *et al.*^46^	1 (0/1)	N/A	0.75 years	Skin (1), lung (1), RP (-), oesophagus (-), heart (1)
Morton and Powell^47^	15 (12/3)	D (8), L (5), O (2) §	7.9 years (<1–27 years)	Skin (15), lung (-), RP (11), oesophagus (10), heart (1)
Nunokawa *et al.*^48^	1 (1/0)	D	5 years	Skin (1), lung (-), RP (1), oesophagus (1), heart (1)
Patrick *et al.*^49^	4 (2/2)	D (3), L (1)	4.5 years (2–8 years)	Skin (4), lung (3), RP (-), oesophagus (4), heart (-)
Quartier *et al.*^50^	4 (4/0)	D §	~0.69 years. (0.3 to ~1.2 years)	Skin (4), lung (3), RP (3), oesophagus (4), heart (4)
Roch *et al.*^51^	1 (1/0)	N/A	1.75 years	Skin (1), lung (1), RP (1), oesophagus (1), heart (-)
Tooze *et al.*^52^	1 (0/1)	N/A	0.5 years	Skin (1), lung (0), RP (0), oesophagus (0), heart (0)
Vayssairat *et al.*^53^	4 (3/1)	N/A	4.5 years (2–7 years)	Skin (4), lung (3), RP (4), oesophagus (3), heart (0)
Wörle *et al.*^54^	4 (4/0)	D (2), L (2) §	9.5 years (1–21 years)	Skin (4), lung (2), RP (4), oesophagus (4), heart (2)
Yamasue *et al.*^55^	1 (1/0)	N/A §	0 years	Skin (1), lung (1), RP (1), oesophagus (1), heart (-)
Yocum and Wilder^56^	1 (1/0)	D	3.5 years	Skin (1), lung (1), RP (1), oesophagus (1), heart (0)
Zachariae and Zachariae^57^	2 (1/1)	D (1), N/A (1)	N/A (1 to several years)	Skin (2), lung (-), RP (1), oesophagus (1), heart (-)
Zachariae *et al.*^58^	10 (7/3)	D (3), L (7) §	11.4 years (1–30 years)	Skin (10), lung (8), RP (-), oesophagus (9), heart (-)
Zachariae *et al.*^59^	1 (1/0)	D	8 years	Skin (1), lung (0), RP (-), oesophagus (1), heart (-)
Zentilin *et al.*^60^	2 (2/0)	N/A	2.75 years (0.5–5 years)	Skin (2), lung (-), RP (2), oesophagus (2), heart (-)

Pat, total patient number; F/M, number of females/number of males; N/A, information not available.

Subtypes: D (diffuse), L (limited), and O (overlap), numbers in parenthesis are the number of patients affected, §: patients fulfilling the ACR criteria.

(-) = not examined/ not specified; (0) = examined and organ manifestation excluded.

a Several patients received medication for RP. However, there was no information provided neither on how many patients nor on the number of patients affected by RP.

b Lung involvement as well as oesophageal and cardiac manifestations and their development during CsA therapy have been observed. However, there was no information on the number of patients affected before initiation of therapy.

Assessment of the disease duration and organ manifestations is presented in Table 2.

Disease duration: A variety of definitions of disease duration were used, with time since diagnosis and time since onset of RP or first non-Raynaud symptom attributable to SSc being the most frequent definitions. Sometimes, there was no indication of the definition of disease duration, and in one article, different information was given for individual patients. Mean disease duration for each paper is mentioned, if parameters for calculation were available or disease duration reported.

Skin: Presence of skin fibrosis was defined as clinical and/or histological evidence of increased skin thickening measured by different skin scores in clinical examination, assessment of physician or patient, plicometry, elastometry, and/or skin biopsy. Clinical examination included the mRSS (estimating skin thickness in 17 body areas by palpation), the University of California at Los Angeles (UCLA) skin score (measuring 10 body areas by clinical palpation), the plicometer skin test (using a plicometer to evaluate nine skin areas), and a skin score without any further information.^33,62,63^

Lung: Pulmonary manifestations were assessed mainly by PFTs (restrictive ventilation disorder, reduced compliance, reduced diffusion capacity of carbon monoxide (DLCO), VC, or FVC). For the purpose of this study, PFTs were considered reduced when showing FVC or DLCO less than 80% of predicted value if no other definition was provided. Several studies referred to reduced lung function without information on the lung function values. Other measures consisted of chest radiography or high-resolution computed tomography (HRCT) revealing changes referred to as interstitial fibrosis, interstitial changes, or as ground-glass opacities and/or indication of pulmonary involvement without further details. In one study, lung findings were assessed also by blood gas analysis and right heart catheter determining PAH.^39^

RP: In one case report, RP was provoked by cold stimulation.^43^ In the remaining studies, there was no indication of whether a cold stimulation test had been performed.

Oesophagus: To detect oesophageal involvement, manometry or radiologic imaging with barium containing contrast medium sometimes combined with 24-h pH-metry was used. If there was no indication of the diagnostic method used, oesophageal involvement was reported as dysphagia, esophagitis, reflux, dysmotility, or dysfunction.

Heart: Cardiac involvement was measured using echocardiography, chest X-ray, and/or electrocardiography.

At the start of therapy, all patients suffered from skin fibrosis and 73 (54.4%) from pulmonary manifestations. Baseline information on renal manifestations was limited and available in only 58 patients (20 studies).

Twenty-eight papers (75.7%) provided information on previous treatments. Previous disease-modifying drugs are listed in the “Treatment regimens” section.

### Treatment regimens

In 34 of 37 papers, patients had CsA treatment. In most studies, dosage was given in mg/kg/d. CsA was applied in dosages between 1 and 10 mg/kg/day body weight (mean 4.4 mg/kg/day). However, the dose was adjusted in the majority of papers during treatment. In three cases, the only information given was in mg daily (range 150–300 mg) and/or as serum concentration.^27,28,35^ CsA was administered over a period of 13 days to more than 5 years, with a mean treatment duration of 15 months.

Twenty-one patients from four studies received Tac: 13 patients in three studies as first-line therapy between 1 and 8 mg daily (mean 2.6 mg/d) and eight patients in one study as second-line treatment.^44,47,48,55^ Treatment duration was between 21 days to 469 weeks (mean 22 months).

Twenty-two papers (59.4%) reported that other immunosuppressive drugs were concomitantly administered in addition to CNI therapy. CNIs were used as first-line therapy in only one study with two patients.^38^ Accordingly, prior application with other agents than CNIs were frequent. Previous potentially disease-modifying drugs included corticosteroids (27 patients), D-penicillamine (24 patients), azathioprine (AZA, 14 patients), methotrexate (MTX, 8 patients), hydroxychloroquine (5 patients), cyclophosphamide (4 patients), colchicine (3 patients), intravenous immune-globulins (3 patients), pentoxifylline (3 patients), and interferon (3 patients). Co-treatments were frequently used including corticosteroids and other immunosuppressive agents (AZA; MTX, prednisone high/low dose (>10 mg/day/⩽10 mg/day), iloprost) (Table 1).

### Effects on skin fibrosis

Therapeutic effects on skin fibrosis over time were observed in 96 (89.7%) patients of 27 studies involving 107 patients, of which eight patients of five studies did receive co-treatments with potentially disease-modifying immunosuppressive drugs. Different methods were used to assess skin fibrosis (Tables 3 and 4).

**Table 3.** Course of skin fibrosis under CNI therapy (without potential disease-modifying immunosuppressive co-treatments).

Publication	Davies and Dunn^35^	Gisslinger *et al.*^39^	Heickendorff *et al.*^40^	Hider *et al.*^41^	Ishida *et al.*^43^	Mehregan and Su^45^	Mérot *et al.*^46^	Morton and Powell^47^	Nunokawa *et al.*^48^	Patrick *et al.*^49^	Tooze *et al.*^52^
Measuring method during process	M	P	M	R	U	M	SS	P	M, B	R	E
Patients [number]	1	9	1	1	10	1	3	10^a^	4	8	5
Improvement [number]	1	7	1	1	6	1	2	N^a^	2	7	5
Unchanged [number]	–	2	–	–	–	–	1	N^a^	–	–	–
Worsening [number]	–	–	–	–	–	–	–	N^a^	–	1	–
Not provided [number]	–	–	–	–	4	–	–	–^a^	2	–	–
Significant improvement	N	S	N	N	S	N	N	S^a^	N	S	S
Publication	Knop and Bonsmann^24^	Ishida *et al.*^43^	Morton and Powell^47^	Patrick *et al.*^49^	Roch *et al.*^51^	Tooze *et al.*^52^	Vayssairat *et al.*^53^	Wörle *et al.*^54^	Yocum and Wilder^56^	Zachariae and Zachariae^57^	Zachariae *et al.*^58^
Measuring method during process	M(1) SS(2)	M	M+	U	M	M	M	M	M, B	M	M
Patients [number]	3	1	15	4	1	1	4	4	1	2	10
Improvement [number]	3	1	9	–	1	1	4	1	1	2	4^b^
Unchanged [number]	–	–	3	4	–	–	–	2	–	–	3^b^
Worsening [number]	–	–	–	–	–	–	–	1	–	–	1^b^
Not provided [number]	–	–	3	–	–	–	–	–	–	–	2^b^
Significant improvement	N	N	N	N	N	N	N	N	N	N	N

Evaluation: M, assessment of a physician; M+, assessment of physician and patient; *R*, RSS; U, UCLA skin score; SS, skin score without any further information; B, biopsy; P, plicometry skin score; E, elastometry.

Numbers in parenthesis are the number of patients affected.

S, significant; *N*, no information on significance available.

a Significant improvement of skin sclerosis in the patient group without information on individual disease course.

b One patient showed improvement without any further information; another showed improvement in digital ulcers without specific information on skin fibrosis.

**Table 4.** Course of skin fibrosis under CNI therapy (with potential disease-modifying immunosuppressive co-treatments).

Publication	Appelboom and Itzkowitch^28^	Constantopoulos *et al.*^34^	Mérot *et al.*^46^	Nunokawa *et al.*^48^	Quartier *et al.*^50^
Measuring method during process	M+	M	M	M	SS
Patients (number)	1	1	1	1	4
Improvement (number)	1	1	1	–	4
Unchanged (number)	–	–	–	–	–
Worsening (number)	–	–	–	1	–
Not provided (number)	–	–	–	–	–
Significant improvement	N	N	N	N	N

Evaluation: M, assessment of a physician; M+, assessment of physician and patient; SS, skin score without any further information.

Numbers in parenthesis are the number of patients affected.

N, no information on significance available.

An improvement of skin fibrosis was experienced by 77 of 96 (80.2%) patients, of which three patients were also taking high-dose PSL and four patients a combination therapy of high-dose PSL and MTX together with CNI. Improvement was described as ‘normalization’, ‘improvement’, or ‘slight improvement’. Endpoints used to assess effectiveness were global physician and patient assessment (33 patients), skin scoring (22 patients), plicometry (17 patients), and elastometry (five patients). Skin biopsies confirmed findings of clinical examinations in two studies (three patients). An unchanged and stable course of skin fibrosis was reported in 15 patients (15.6%) within six studies. Assessments were done by global physician/patient assessment (eight patients), skin scoring (five patients), and plicometry (two patients). Worsening of skin fibrosis was observed in four patients (4.2%) from four studies, one receiving concomitant therapy with high-dose PSL. Three patients deteriorated according to global physician assessment, and one of them reported worsening of mRSS.

In four patients of three studies, CsA was withdrawn after remission of skin fibrosis and the clinical course was reported after withdrawal.^25,29,30^ Three patients relapsed, one within 6 months, and CsA had to be re-started in all three patients.^29,30^ The fourth patient remained in remission.^25^

Because of the spontaneous regression of skin fibrosis often seen in patients with SSc, treatment studies without control groups must be interpreted with caution.^24^ We therefore report on the two studies with control groups in more detail. Both studies observed a significant improvement of skin fibrosis under CsA therapy. Clements *et al.*^33^ performed an open study with 10 patients under CsA treatment and compared the effects with a historical control group. The initial dose of 1 mg/kg/d CsA was increased to 5 mg/kg/d if tolerated. After 48 weeks of therapy, the UCLA skin score improved significantly *versus* baseline (*p* < 0.0001) and its improvement was significantly higher (*p* < 0.004) than in the historical control group. Six of the 10 patients had an improvement of skin score greater than 35%. In the randomized controlled study of Filaci *et al.*,^37^ the effects of CsA at a dose of 2.5 mg/kg/d *versus* no treatment were assessed in 20 patients. All patients received therapy with iloprost. After 12 months, the treatment group showed a significant decrease of the plicometry skin score (*p* = 0.008), while the score of the no-treatment control group decreased without significance (*p* = 0.1).

### Effects on lung manifestations

Lung involvement at therapy start was reported in 29 studies. Out of 96 patients, 74 (77.0%) had SSc-associated lung manifestations.

Pathological PFTs were reported in 59 (61.5%) patients. Of these patients, 10 had a forced vital capacity (FVC) below 80% and 4 below 70% predicted. Twenty-nine patients had ILD confirmed by chest radiography or HRCT. Twenty-three of these patients had both altered PFTs and interstitial changes on imaging.

PAH was diagnosed in six patients of two studies, one study with five patients using a right heart catheter for diagnosis, while for the sixth patient, the definition of PAH diagnosis was not provided.^39,44^ Finally, pulmonary involvement was mentioned in seven patients without further details.

We next assessed changes under treatment based on their outcome measures. For PFTs, the following changes were reported (Tables 5 and 6, *n* = 22 studies). In 28 patients, the course of pathological PFTs have been described individually, improving in 19, remaining stable in eight and aggravating in one patient. Another patient did not have pulmonary manifestations at the beginning but rather developed them during CsA therapy.^35^ Of these patients, six had been receiving high-dose PSL alone or in combination with MTX concomitantly to CNI therapy. In the retrospective study including 11 patients with interstitial pneumonia treated with Tac and high-dose PSL, changes in PFTs were assessed in the whole patient group without information on the individual disease course.^44^ After 1 year of Tac administration, pulmonary function did not significantly change on the group level.

**Table 5.** Development of pulmonary manifestations under CNI therapy (without disease-modifying immunosuppressive co-treatments).

Study	Ando *et al.*^27^	Basso *et al.*^29^	Basso *et al.*^30^	Casoli *et al.*^31^	Clements *et al.*^33^	Davies and Dunn^35^
Number Pat.	1	9	1	1	10	1
Lung findings before therapy	1 (L, H)	7 (L)	1 (L)	1 (L, T)	4 (T), 3 (K)	0 (T, L)
Improvement	**1 (L)**	**7** ^s^ **(L)**	**1** ^a^ **(L)**	**1 (L, T)**	–	–
Unchanged	**1 (H)**	*2*	–	–	**4 (T)**	–
Deterioration	–	–	–	–	–	*1 T, L*
Study	Filaci *et al.*^37^	Francès *et al.*^38^	Gisslinger *et al.*^39^	Ippoliti *et al.*^42^	Ishida *et al.*^43^	Roch *et al.*^51^
Number Pat.	10	4	8	5	1	1
Lung findings before therapy	N.A.	2 (L)	4 (T), 5 (P)	4 (K)	1 (L)	1 (L)
Improvement	–	–	**5 (P)**	**4 (L)** ^b^	**1 (L)**	**1 (L)**
Unchanged	*10* ^ns^ *L*	**2 (L)**	**3 (T)**	–	–	–
Deterioration	–	–	**1 (Pn)**, **1 (T)**	–	–	–
Study	Vayssairat *et al.*^53^	Wörle *et al.*^54^	Yamasue *et al.*^55^	Yocum and Wilder^56^	Zachariae and Zachariae^57^	
Number Pat.	4	4	1	1	2	
Lung findings before therapy	3 (L, T)	2 (L^c^)	1 (L, H)	1 (L)	N.A.	
Improvement	–	**1 (L** ^c^ **)**	–	–	**1** ^d^ **(L)**	
Unchanged	**3 (L, T)**	**1 (L)**	–	**1 (L)**	–	
Deterioration	–	–	**1 (L, H)**	–	–	

a The line ‘lung findings’ shows the number of patients with pulmonary involvement and their corresponding pathology before CNI therapy. These patients are documented with highlighted (bold) writing. Narrow, italic numbers stand for the course of patients without lung manifestation before therapy (if documented).

L, pathological lung function (in spirometry or Dlco); T, pulmonary manifestation on chest radiography; H, pulmonary manifestation in HRCT; P, PAH; Pn, PAH developed under CsA therapy; K, indication of pulmonary involvement, but neither diagnosis nor explicit disease course was available.

s Significant improvement of the mean lung function score after 3 years compared to the start of therapy (*p* = 0.03).

ns Non-significant change in the patient population and no information on the individual patient course.aImprovement of organ manifestations was reported without specific mention of pulmonary involvement.

b Partial recovery of lung function in the patient population without further details.

c An improvement in respiratory function has been reported in one patient with no indication of whether PFTs have been performed or not.

d Improvement of FEV1 from 1.7 to 1.9 L in lung function without further details.

**Table 6.** Development of pulmonary manifestations under CNI therapy (with potential disease-modifying immunosuppressive co-treatments).

Study	Appelboom and Itzkowitch^28^	Constantopoulos *et al.*^34^	Hider *et al.*^41^	Konma *et al.*^44e^	Quartier *et al.*^50^
Number Pat.	1	1	1	11	4
Lung Findings before therapy	1 (L)	1 (L, T)	1 (L, H)	10 (L), 11 (H)	3 (L)
Improvement	1 (L)	1 (L, T)	**1 (L)**	**3**	**2 (L)**
Unchanged	–	–	**1 (H)**	**7**	**1 (L)**
Deterioration	–	–	–	1	–

The line ‘lung findings’ shows the number of patients with pulmonary involvement and their corresponding pathology before CNI therapy. These patients are documented with highlighted (bold) writing. Narrow, italic numbers stand for the course of patients without lung manifestation before therapy (if documented).

L, pathological lung function (in spirometry or Dlco); T, pulmonary manifestation on chest radiography; H, pulmonary manifestation in HRCT.

a Therapy effectiveness of Tac was assessed according to the ATS/ERS guidelines using three criteria (decrease in symptoms, reduction of abnormalities on HRCT, and improved lung function). Treatment response was defined as ‘improved response’, ‘stable response’, or ‘failure to respond to therapy’ according to which of the criteria were met. It was not possible to draw conclusions about individual changes in the three criteria.

Studies also reported changes on imaging outcomes: Of the 29 patients using X-ray or HRCT to evaluate interstitial changes, information on the individual lung changes over time was available in 16 cases (55.2%). Over the course of therapy, interstitial changes improved in two, remained stable in 12 and worsened in another two patients. The same patient who developed pathological lung function under therapy also developed new pulmonary fibrosis on chest X-ray.^36^ The retrospective study reported a decrease in the total fibrosis score in HRCT after 1 year of Tac but without significance, while the total ground-glass opacity (GGO) score showed a significant decrease (*p* = 0.005).

Combined outcomes measures were also reported in some studies: A detailed evaluation of the lung findings in eight SSc patients was performed by Gisslinger *et al.*^39^ The lung findings were assessed by blood gas analysis, chest X-ray, and PFTs at baseline and after 6 months of CsA therapy. Before therapy, seven of the eight patients showed pathological lung findings: Pulmonary hypertension (five patients), ILD on chest X-ray (four patients), hypoxemia (four patients), and a VC ⩽ 80% predicted (four patients). PAH improved in all five affected patients, but another patient newly developed PAH during CsA therapy. One of the five PAH patients died 9 months after starting therapy despite improvement in PAH. The autopsy showed fibrinous pericarditis. Four of the five patients with improved PAH under CsA therapy received interferon-2 as additional drug and two patients received 2 mg PSL daily. Interstitial fibrosis on chest X-ray worsened in one of the four patients, while it remained unchanged in the other three. Hypoxemia improved in all four patients. FVC normalized to >80% in two patients, while it remained <80% in the other two.

### Adverse events during CNI therapy

Renal adverse events were reported in 58 patients (43.3%, Tables 7 and 8). Seven patients experienced severe renal complications: one with confirmed hemolytic uremic syndrome (HUS) after combination therapy with CsA and high-dose PSL; one with HUS rather than renal crisis, but a differentiation was not clearly possible; one with SRC, developed 16 days after withdrawal from CsA and was confirmed histologically after death; two with renal crisis or CsA renal toxicity which could not be differentiated; one with SRC confirmed by corresponding renal biopsy; and finally one patient dying from renal insufficiency without the cause of renal insufficiency being identified.^31–33,38,48,54,59^ The raised serum creatinine of these patients was measured between 200 and 530 µmol/L.

**Table 7.** Kidney function under CNI therapy (without potential disease-modifying immunosuppressive co-treatments).

Study	Amor and Dougados^26^	Casoli *et al.*^31^	Chen *et al.*^32^	Clements *et al.*^33^	Denton *et al.*^36^	Francès *et al.*^38^	Gisslinger *et al.*^39^	Heickendorff *et al.*^40^
Patients	2	1	1	10	3	4	8	6
Patients with renal side effects^R^	2	1	1	8	3	1	4	6
Severe complications	–	N1	N2	N3 (1)	–	N4	–	–
Renal biopsy	M, F, I, A (1)	R	D	–	*T* (2)	–	–	sf (2)
Cr increase^C^	2	1	1	8	3	N/A	4	6
Cr increase >30%	2	N/A	1	8	N/A	N/A	4	N/A
Highest Cr^S^/ lowest Cr clearance	517; 330 μmol/L	220^S^ μmol/L	~440^S^ μmol/L	70.4–238^S^ μmol/L (mean 123.2)	3*; 21; 28 mL/min*	N/A	124– 150 μmol/L	N/A
Proteinuria	N/A	N/A	1	N/A	N/A	N/A	N/A	N/A
Haematuria	N/A	N/A	1	N/A	N/A	N/A	N/A	N/A
Hypertension	2	1	1	2	3	N/A	N/A	N/A
Highest blood pressure	200/130; 180/110	190/120	170/100	>140/90	240/130, N.A. (2)	N/A	N/A	N/A
Study	Knop and Bonsmann^24^	Ippoliti *et al.*^42^	Ishida *et al.*^43^	Morton and Powell^47^	Vayssairat *et al.*^53^	Wörle *et al.*^54^	Zachariae *et al.*^58^	Zentilin *et al.*^60^
Patients	3	5	1	15	4	4	10	2
Patients with renal side effects^R^	1	5	1	2	1	3	3	2
Severe complications	–	–	–	–	–	N6 (1)	–	–
Renal biopsy	–	–	–	–	–	–	AP (2)	–
Cr increase^C^	1	**	b	2	1	3	1	2
Cr increase >30%	N/A	N/A	N/A	N/A	N/A	1	–	–
Highest Cr/ lowest Cr clearance	N/A	N/A	N/A	N/A	135 μmol/L	528^S^, ~132^S^, ~79^S^ μmol/L	N/A	N/A
Proteinuria	N/A	N/A	N/A	N/A	N/A	N/A	N/A	N/A
Haematuria	N/A	N/A	N/A	N/A	N/A	N/A	N/A	N/A
Hypertension	N/A	2	N/A	N/A	N/A	N/A	N/A	N/A
Highest blood pressure^BP^	N/A	N/A	N/A	N/A	N/A	N/A	N/A	N/A

Articles with reports on renal side effects are listed.

Numbers in parenthesis are the number of patients affected.

-, renal side effects did not occur; N/A, no information provided.

R In numerous studies, no definition was provided on what was understood to be a renal complication. For the purpose of this study, all patients showing Cr increase above or Cr clearance below baseline during CNI treatment, pathological kidney biopsy consistent with SRC, SSc or CsA-arteriolopathy and b2-microglobulin as sign of renal damage were regarded to suffer from renal adverse events. In two articles, serum Cr was considered abnormal only with values below 1.2 mg/dL (105.6 μmol/L) or 125 μmol/L, respectively.^32,39^

C Increase of Cr or decrease of Cr clearance.

S If the information on serum Cr was provided in mg/mL or mg/dL, it was converted into µmol/L (mg/dL = 88.4 μmol/L).

β, increase of urinary b2-microglobulin without any further information or adverse events.

BP Only one study defined hypertension as having a diastolic blood pressure over 90 mmHg, all the other articles dealing with patients having hypertension did not provide any definitions.^39^

N1: SRC (sudden onset of renal failure and malignant hypertension) developed 16 days after CsA withdrawal and was histologically confirmed after death following haemorrhagic insult.

* One patient had a severe loss of renal function after withdrawal from CsA.

** A significant decrease of Cr clearance was shown without any further manifestations or complications.

**Table 8.** Kidney function under CNI therapy (with potential disease-modifying immunosuppressive co-treatments).

Study	Appelboom and Itzkowitch^28^	Konma *et al.*^44***^	Nunokawa *et al.*^48^	Zachariae *et al.*^59^
Patients	1	11	1	1
Patients with renal side effects^R^	1	11	1	1
Severe complications	–	–	N5	N7
Renal biopsy	–	–	R	M
Cr increase^C^	1	11	1	1
Cr increase >30%	N/A	N/A	N/A	1
Highest Cr^S^/lowest Cr clearance	29 mL/min	45.8^S^–108.2^S̈^μmol/L	530^S^ μmol/L	201 μmol/L
Proteinuria	N/A	N/A	1	1
Haematuria	N/A	N/A	1	1
Hypertension	–	N/A	1	1
Highest blood pressure	–	N/A	174/72	180/110

Articles with reports on renal side effects are listed.

Numbers in parenthesis are the number of patients affected.

-, renal side effects did not occur, N/A = no information provided.

R In numerous studies, no definition was provided on what was understood to be a renal complication. For the purpose of this study, all patients showing Cr increase above or Cr clearance below baseline during CNI treatment, pathological kidney biopsy consistent with SRC, SSc or CsA-arteriolopathy and b2-microglobulin as sign of renal damage were regarded to suffer from renal adverse events. In two articles, serum Cr was considered abnormal only with values below 1.2 mg/dL (105.6 μmol/L) or 125 μmol/L, respectively.^32,39^

C Increase of Cr or decrease of Cr clearance.

S If the information on serum Cr was provided in mg/mL or mg/dL, it was converted into µmol/L (mg/dL = 88.4 μmol/L).

β, increase of urinary b2-microglobulin without any further information or adverse events.

N1: SRC (sudden onset of renal failure and malignant hypertension) developed 16 days after CsA withdrawal and was histologically confirmed after death following haemorrhagic insult.

*** Renal side effects after one year of Tac administration.

An increase of serum creatinine or a decrease in creatinine clearance without severe renal side effects was detected in 48 patients, in 31 of 48 patients, serum creatinine ranging from >125 to <300 µmol or creatinine clearance decreasing on values <30 mL/min, respectively.^26,28,33,36,39,44,53,54^ In 17 of the 48 patients, creatinine/creatinine clearance was not quantified. Another three patients with renal side effects had no information on creatinine/creatine clearance reported. One of them had an increase of urinary β2-microglobulin without any other diagnosis or information, while the other two showed pathologic renal biopsy with CsA-induced arteriolopathy, one of them having just a slightly changed histology.^43,58^

Altogether, hypertension was noticed in 13 patients.^26,31–33,36,42,48,59^ Pathological renal biopsy was reported in 11 patients.^26,31,32,36,40,48,58,59^

Next, we assessed, how often adverse events led to discontinuation of CNI therapy. In total, 39 of 134 (29.1%) patients showed considerable renal but also extra-renal adverse events, leading to reduction (five cases), interruption, or withdrawal (29 cases) from CNI therapy or death (five cases). Besides, severe kidney adverse events (7 cases), neurological adverse events (six cases), and insufficient therapy of CsA (six cases) were among the most frequent causes for discontinuation. In one patient, the immunosuppressive therapy led to exacerbation of autoimmune pulmonary alveolar proteinosis and development of adenocarcinoma of the lungs.^55^ Death occurred in five cases during CsA therapy or soon after withdrawal.^38,39,50^ Three patients died of cardiac adverse events (pericarditis, fibrosis, and heart insufficiency), one suffered from acute renal insufficiency and in one case no information on the cause of death was provided.

## Discussion

### Important results

This systematic literature review contains the findings of 37 articles, two of them being controlled studies: one with a prospective randomized no-treatment control group and another with a retrospective historical control group.^33,37^ Overall, 134 SSc patients treated with CNIs were identified from all studies, most of them suffering from dcSSc, which is the classical indication to apply potentially disease-modifying agents.

The most frequently used CNI was CsA with dosages varying between 1 and 10 mg/kg body weight (mean 4.4 mg/kg) or 150–300 mg/d, respectively. Tac was applied between 0.03 and 0.18 mg/kg daily (mean 0.04 mg/kg) or 1–8 mg daily (mean 2.6 mg/d). These wide dose ranges indicate the lack of consensus regarding CNI therapy and its optimal treatment dosage. Limited data are available for the treatment of SSc with Tac because it has only been used in four of the 37 papers.

In most of the papers, CNIs were used as a second-line therapy. Out of the 28 papers (75.7%) providing information about previously used agents, only two patients from one study reported receiving CNIs as a first-line treatment. This implicates that many patients already had ineffective previous therapies, suggesting severe and treatment-resistant forms of SSc, which needs to be taken into account when interpreting treatment effectiveness for CNIs. Furthermore, as most patients identified in this systematic literature review suffered from the dcSSc (53%) which is a known risk factor for SRC, the prevalence of renal insufficiency and kidney-related adverse events have to be interpreted with caution.^11^ The combination of potentially nephrotoxic CNIs in patients at risk for renal complications might not be representative for the overall SSc population. Another important point to consider when interpreting the result is the long disease duration with a mean of 5.8 years (range 0–30 years). In these later stages of SSc, the disease course is getting less dynamic, and treatment effects are more difficult to show because of the overall more stable natural course.

### Therapy results

Given the limitations mentioned above and the general limitations of interpreting effectiveness from literature reviews, this study provides signals for potential effectiveness of CNIs to treat skin fibrosis in SSc patients. Most patients (80.2%) had an improvement of fibrosis, among them the patients in both studies with control groups, showing a significant reduction of skin fibrosis under CsA therapy.^33,37^ Unfortunately, the quality of the control groups was limited consisting of a historical control group and a no-treatment control group, respectively. A parallel placebo or effective treatment control group would be required to reach a higher level of evidence, particularly in SSc, where spontaneous regression of skin fibrosis under a standard of care is frequent.^64^ However, potential effectiveness of CNIs is further underlined by the finding that 18 articles showed a clinically important improvement of skin fibrosis.

CNIs could also have a positive effect on pulmonary fibrosis. Pulmonary function improved in almost 70% of the patients (19/28) who were individually assessed during the studies. In contrast to PFTs, imaging showed no improvement in most patients (14/16) during CNI therapy. This might not be surprising considering the low sensitivity to change of qualitative assessment of imaging and X-rays specifically. Indeed, HRCT was performed in four case reports only. The remaining studies used chest X-rays for diagnosis and evaluation of the course of pulmonary fibrosis. To better assess the effect on pulmonary fibrosis, a longitudinal, prospective, randomized, controlled study with sufficient treatment duration should be conducted.

PH, which was present in six patients at baseline, was assessed in five patients at follow-up, reporting improvement in all patients.^39^ Another patient developed a new PAH under CsA therapy.^39^ However, due to these limited numbers, results from an open pilot study, the lack of a control group and the old definitions used for PAH at the time of the study, it is impossible to draw any conclusions on effectiveness for this indication. In addition, immunosuppressive drugs in general have not been confirmed to be an effective treatment strategy in patients with SSc-PAH.

### Adverse events and complications

Severe renal side effects were reported in almost 10% of the patients included in this review, which is beyond what would be expected for such a population. Also considering the established nephrotoxicity of CNIs, therapy with CNIs should be conservatively selected and preferentially used in patients without risk factors for renal involvement. Renal toxicity of CsA has been shown to be dose-dependent.^65^ Therefore, high doses should be avoided until more robust RCT data with adequate control groups are available for effectiveness and (renal) toxicity. This statement is further supported by our data. Clements *et al.*^33^ reported that renal adverse events during therapy of SSc with CsA occur frequently with doses >3–4 mg/kg/day. Patients with severe renal adverse events had a dose range of CsA from 2.2 to 10 mg/kg, only 1 of 7 having a maximal dose lower than 3 mg/kg CsA per day.^54^

Therapy withdrawal (29 cases), treatment interruption (five cases), or death (five cases) following side effects were frequent (31.7%). The main reasons were neurological (six patients) or renal manifestations (thirteen patients), which in one patient even ended fatally due to acute renal insufficiency. Despite good blood monitoring of CsA and early detection of nephrotoxicity, renal crisis occurred in three cases.^36^

Interestingly, in an attempt to reduce side effects, a second-generation CNI, voclosporin, has been introduced more recently. Voclosporin has a higher potency than CsA requiring a 10 times lower dosage than CsA. It appears to be better tolerable and less nephrotoxic.^66^ This has been confirmed in phase II and phase III RCTs for chronic plaque psoriasis.^67^ However, while there was no increase in Cr levels at low-dose voclosporin (0.5 mg/kg/d), a significant increase of serum Cr was shown in the patient group treated with voclosporin 1.5 mg/kg/d compared to the placebo group, but the Cr values remained within normal range. A phase III, study reported on a slight reduction of GFR in eight out of 451 patients with plaque psoriasis, but no clinically significant renal toxicity.^68^ Whether these findings with lower toxicity are also holding true for a patient population with pre-existing risk for renal impairment like SSc, needs to be analysed in appropriate trials.

### Strengths and limitations of this review

This review was performed systematically with predefined selection criteria. Consequently, this publication is the largest review about the treatment and side effects of CNIs in SSc patients.

The most important limitation of this review is the heterogeneity of the available data. Numerous different outcome measures, inclusion criteria, and classification criteria were used. In addition, a variety of treatment regimens and dosages were applied, and most papers used retrospective, uncontrolled settings with small numbers of patients, making reliable conclusions on effectiveness and toxicity challenging.

## Conclusion

In conclusion, this systematic literature review provides signals for a potential effectiveness of CNIs and particularly CsA for the treatment of skin and pulmonary fibrosis in SSc. However, the evidence level of the identified studies was low and the number of patients treated in the various studies was small, making reliable conclusions on effectiveness challenging. This shows the importance to conduct a larger, prospective randomized, double-blinded controlled trial to assess the effectiveness of CNIs on SSc patients.

The general dose-dependent nephrotoxicity of CNIs, which was confirmed in this study, has to be considered when designing such RCTs. This could be addressed by excluding patients at risk for SSc-associated renal manifestations from these trials and by closely monitoring renal toxicity in the trial. In addition, due to its reduced toxicity compared to CsA, voclosporin could be a promising candidate to be used in such RCTs.

## Supplemental Material

sj-docx-1-tab-10.1177_1759720X221092374 – Supplemental material for Calcineurin inhibitors in systemic sclerosis – a systematic literature review Click here for additional data file. Supplemental material, sj-docx-1-tab-10.1177_1759720X221092374 for Calcineurin inhibitors in systemic sclerosis – a systematic literature review by Nina N. Hofmann, Robert A. Ambühl, Suzana Jordan and Oliver Distler in Therapeutic Advances in Musculoskeletal Disease
